# Migrated fish bone induced liver abscess: medical management

**DOI:** 10.11604/pamj.2020.36.140.23783

**Published:** 2020-06-30

**Authors:** Moustafa Allam, Stephanos Pericleous

**Affiliations:** 1 Centre of HPB Surgery and Liver Transplantation, Royal Free Hospital, London, United Kingdom

**Keywords:** Liver abscess, foreign body perforation, fish bone, medical treatment, case report

## Abstract

Liver abscess secondary to a migrated ingested foreign body is an uncommon condition where early diagnosis helps management and improves prognosis. Abscess drainage with removal of the foreign body is the recommended management. We report the successful management of a patient with a liver abscess from a migrated fishbone that was treated medically with the foreign body left in situ.

## Introduction

Pathogens that spread through the portal vein from the gastrointestinal tract (GIT), the hepatic artery from systemic sepsis, or the bile duct as in ascending cholangitis, are all recognized causes of liver abscesses [1]. Perforation of hollow viscus by a foreign body (FB) is rare, representing 1% of cases of accidental foreign body ingestion. However, development of a liver abscess secondary to penetrated FB is even less common. Reports of ingested foreign bodies causing liver abscesses have become more frequent especially in cases where abscesses fail to resolve [1,2]. We present a case of a complex liver abscess caused by a migrated fishbone which was managed without invasive procedures with eventual complete resolution.

## Patient and observation

**Figure 1:**
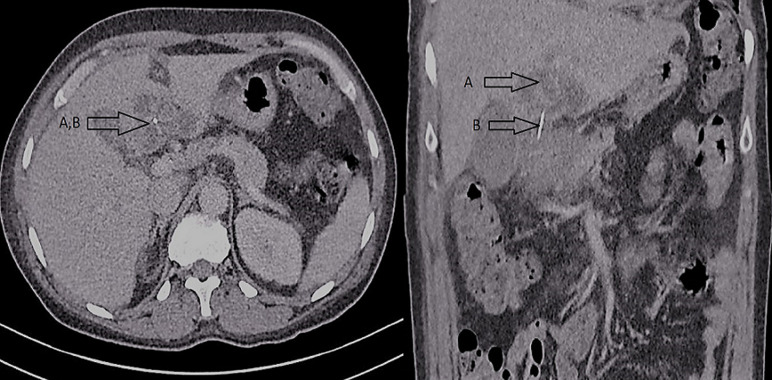
CT scan performed on admission, showing the septated segment III/IVb liver abscess (A) and the fishbone (B)

**Figure 2:**
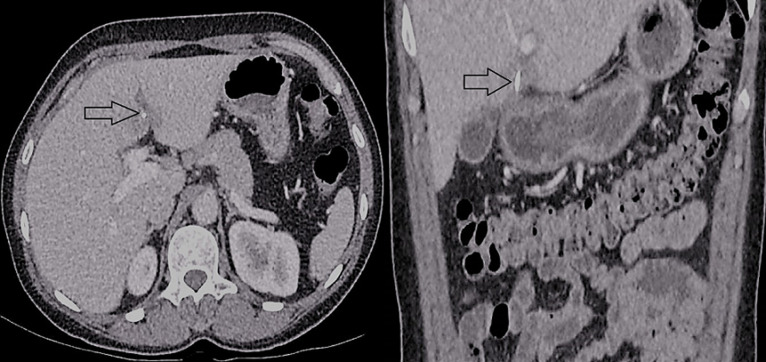
CT scan 6 weeks post presentation with resolving liver abscess and fishbone remains in place as indicated by arrows

A 53-year-old female presented to the emergency department with a 1-week history of dull progressive lower abdominal pain radiating to the upper abdomen and back. The patient reported fevers and shivering. She had a past medical history of irritable bowel syndrome, depression and had an open appendicectomy at the age of 17. On examination, the patient had a fever at 38^o^C and a heart rate of 92/min. Her abdomen was tender throughout, markedly in the right upper quadrant. Blood tests showed leukocytosis at 14.8 10^9^/L (Normal value: 4-11 10^9^/L), high c-reactive protein at 253mg/L (Normal value: 0-5mg/L) with normal amylase level at 18U/L (Normal value: 30-118U/L). A computed tomography (CT) scan done on admission, showed an ingested FB perforating through the pylorus, embedded in the liver with adjacent mesenteric stranding and a 5.6cm loculated liver abscess transgressing segments III/IVb (Figure 1). It then transpired that the patient had inadvertently swallowed a fishbone approximately one week prior to presentation. The abscess was not amenable to drainage owing to its multilocularity. Hence, she was managed conservatively as an inpatient with a month´s therapy of intravenous antibiotics and received piperacillin tazobactam 4.5grams four times daily and metronidazole 500mgs three times daily. In addition, total parenteral nutrition was started, whilst the patient was nil by mouth. Following satisfactory clinical and radiological improvement the patient was discharged home with outpatient follow-up. Six weeks follow up CT scan (Figure 2) demonstrated replacement of the complex liver abscess with a small focus of low attenuation change on either side of the falciform ligament and the linear foreign body extending from the pyloric wall into segment four of the liver remained unchanged in position. On her annual follow up, the patient reported being completely asymptomatic. An ultrasound scan (US) confirmed complete resolution of the abscess, but the fishbone was still visible. She was discharged from routine follow-up.

## Discussion

A liver abscess as a result of FB migration from the GIT was first described by Lambert in 1898 [2]. This diagnosis can often be mislabeled as a cryptogenic liver abscess despite a full clinical and radiological assessment with CT and US [3]. To date there is little consensus on the management of liver abscesses secondary to migrated FB. The first step is to establish a firm diagnosis; however, treatment failure is usually down to misdiagnosis. A retained FB should be suspected in cases where a left lobe liver abscess fails to resolve with standard treatment especially in the absence of underlying risk factors [3]. Surprisingly, such patients rarely give a history of an ingested foreign body. In the literature, only 5% of patients recalled ingesting a FB. In terms of the type of ingested FB, fish bones is the commonest reported (33%), followed by toothpicks (27.3%), chicken bones (12.5%) and needles (9.1%) [1,3]. Clinical presentation for hepatic abscess in patients with migrating FB is often vague and non-specific, however, abdominal pain (77.3%) and fever (58%) are the most common presenting complaints [1,4]. Occasionally, FB impaction and perforation can present as an acute upper gastrointestinal bleed[3]. Contrast enhanced CT scanning is the standard diagnostic modality offering a high resolution and accuracy, followed by abdominal ultrasonography [1,5].

The most common site of FB perforation is the stomach (40.9%) with the formation of left liver lobe abscess. Duodenal and colonic perforations have also been reported in the literature [1]. The most frequently isolated organisms in these abscesses are streptococci [1]. Isolation of streptococcus milleri on blood cultures is uncommon and should raise the suspicion of an underlying abscess [5]. Other organisms such as *Escherichia coli* and *Klebsiella pneumonia* have also been reported [1]. Most of the published literature recommends removal of the offending FB with abscess drainage as the treatment of choice. This can be achieved through a variety of approaches ranging from interventional radiology (image guided) approach to laparoscopic or formal, open surgery. However, 9.5% of cases can resolve with the FB left in place [1,4-9], whilst 6.8% reported to successfully manage the liver abscess with medical treatment only in the form of antibiotics [1,4,6-8]. In our case, the diagnosis was established on presentation with the reference CT scan. Although US-guided drainage of the liver abscess was considered, the approach was not employed due to the complex nature and location of the septated abscess. The fishbone was left in situ with close observation and follow up. The liver abscess was treated conservatively with antibiotics with complete resolution after one-year follow-up.

## Conclusion

In conclusion, hepatic abscesses due to migrated foreign bodies such as fish bones can be successfully managed conservatively in the right setting. Early accurate diagnosis is paramount, as is diligent clinical and radiological follow up.
